# EPI-Net One Health reporting guideline for antimicrobial consumption and resistance surveillance data: a Delphi approach

**DOI:** 10.1016/j.lanepe.2022.100563

**Published:** 2022-12-22

**Authors:** Nithya Babu Rajendran, Fabiana Arieti, Carla Alejandra Mena-Benítez, Liliana Galia, Maela Tebon, Julio Alvarez, Beryl Primrose Gladstone, Lucie Collineau, Giulia De Angelis, Raquel Duro, William Gaze, Siri Göpel, Souha S. Kanj, Annemarie Käsbohrer, Direk Limmathurotsakul, Estibaliz Lopez de Abechuco, Elena Mazzolini, Nico T. Mutters, Maria Diletta Pezzani, Elisabeth Presterl, Hanna Renk, Jesús Rodríguez-Baño, Oana Săndulescu, Federico Scali, Robert Skov, Thirumalaisamy P. Velavan, Cuong Vuong, Evelina Tacconelli, EPI-Net One Health consensus working group, Ayola Akim Adegnika, Lisa Avery, Marc Bonten, Alessandro Cassini, Claire Chauvin, Monica Compri, Peter Damborg, Sabine De Greeff, Maria Dolores Del Toro, Matthias Filter, Alison Franklin, Bruno Gonzalez-Zorn, Kari Grave, Didier Hocquet, Ludwig E. Hoelzle, Erta Kalanxhi, Ramanan Laxminarayan, Leonard Leibovici, Surbhi Malhotra-Kumar, Marc Mendelson, Mical Paul, Cristina Muñoz Madero, Rita Murri, Laura J.V. Piddock, Carolien Ruesen, Maurizio Sanguinetti, Thorben Schilling, Remco Schrijver, Mitchell J. Schwaber, Luigia Scudeller, Didem Torumkuney, Thomas Van Boeckel, Wannes Vanderhaeghen, Andreas Voss, Teresa Wozniak

**Affiliations:** a Infectious Diseases, Department of Internal Medicine I, University Hospital Tübingen, Tübingen, Germany; b Infectious Diseases Section, Department of Diagnostics and Public Health, University of Verona, Verona, Italy; c VISAVET Health Surveillance Center and Department of Animal Health, Faculty of Veterinary Medicine, Complutense University, Madrid, Spain; d German Centre for Infection Research (DZIF) Clinical Research Unit for Healthcare Associated and Antibiotic Resistant Bacterial Infections, Tübingen, Germany; e French Agency for Food, Environmental and Occupational Health and Safety, ANSES, Maisons-Alfort, France; f Dipartimento di Scienze Biotecnologiche di base, Cliniche Intensivologiche e Perioperatorie, Universita Cattolica del Sacro Cuore, Rome, Italy; g Unit for the Prevention and Control of Infection and Antimicrobial Resistance, Centro Hospitalar do Tâmega e Sousa, Penafiel, Porto, Portugal; h The European Centre for Environment and Human Health, University of Exeter Medical School, University of Exeter, Penryn, Cornwall, UK; i Department of Internal Medicine, Division of Infectious Diseases, Infection Control Program, Antimicrobial Stewardship Program, American University of Beirut Medical Center, Beirut, Lebanon; j German Federal Institute for Risk Assessment (BfR), Department 4 - Biological Safety, Berlin, Germany; k Mahidol Oxford Tropical Medicine Research Unit and Department of Tropical Hygiene, Faculty of Tropical Medicine, Mahidol University, Thailand; l Centre for Tropical Medicine and Global Health, Nuffield Department of Medicine, University of Oxford, UK; m Department of Epidemiology, Istituto Zooprofilattico Sperimentale delle Venezie, Udine-Padova, Padua, Italy; n Institute for Hygiene and Public Health, Bonn University Hospital, Bonn, Germany; o European Committee on Infection Control, Basel, Switzerland; p Department of Infection Control and Hospital Epidemiology, Medical University of Vienna, Vienna, Austria; q ESCMID Study Group for Nosocomial Infections, Basel, Switzerland; r Department of Paediatric Cardiology, Pulmology and Intensive Care Medicine, University Children's Hospital Tübingen, Tübingen, Germany; s Infectious Diseases and Microbiology Division, Hospital Universitario Virgen Macarena/Department of Medicine, School of Medicine, University of Seville/Biomedicine Institute of Seville (IBiS)/CSIC, Seville, Spain; t CIBERINFEC, Instituto de Salud Carlos III, Madrid, Spain; u Department of Infectious Diseases I, Carol Davila University of Medicine and Pharmacy, Bucharest, Romania; v National Institute for Infectious Diseases “Prof. Dr. Matei Balș”, Bucharest, Romania; w Istituto Zooprofilattico Sperimentale della Lombardia e Dell’Emilia Romagna, Brescia, Italy; x Epidemiological Infectious Disease Preparedness, Statens Serum Institut, Copenhagen, Denmark; y Institute of Tropical Medicine, Universitätsklinikum Tübingen, Tübingen, Germany; z Vietnamese – German Center for Medical Research, Hanoi, Vietnam; aa AiCuris Anti-infective Cures GmbH, Wuppertal, Germany; ab Jansen Pharmaceuticals, Beerse, Belgium

**Keywords:** One Health, Antibiotic policy, Antimicrobial resistance, Surveillance, Antimicrobial consumption

## Abstract

Strategic and standardised approaches to analysis and reporting of surveillance data are essential to inform antimicrobial resistance (AMR) mitigation measures, including antibiotic policies. Targeted guidance on linking full-scale AMR and antimicrobial consumption (AMC)/antimicrobial residues (AR) surveillance data from the human, animal, and environmental sectors is currently needed. This paper describes the initiative whereby a multidisciplinary panel of experts (56 from 20 countries—52 high income, 4 upper middle or lower income), representing all three sectors, elaborated proposals for structuring and reporting full-scale AMR and AMC/AR surveillance data across the three sectors. An evidence-supported, modified Delphi approach was adopted to reach consensus among the experts for dissemination frequency, language, and overall structure of reporting; core elements and metrics for AMC/AR data; core elements and metrics for AMR data. The recommendations can support multisectoral national and regional plans on antimicrobials policy to reduce resistance rates applying a One Health approach.

## Introduction

Antimicrobial resistance (AMR) is a critical multifaceted issue that involves humans, animals, and the environment, and should be addressed not only at the level of each individual sector but also where the three sectors interface. Numerous evidence supports that to minimise the emergence and transmission of AMR, it is crucial not only to improve infection prevention and control in the healthcare and veterinary setting, but also to ensure access to clean water, sanitation, and hygiene in healthcare facilities and in the community at large. A One Health approach to surveillance should include all components necessary to identify (new) emerging resistant microorganisms, inform about risk factors and cross-sectoral transmission dynamics, develop and implement relevant antibiotic stewardship interventions, orient patient treatment, and promote policy development in all three sectors to combat AMR.^1^^,^^2^ To efficiently apply such a holistic approach to AMR surveillance is of paramount importance to mitigate the clinical burden of AMR and allow better economic assessment of the problem.^3^ In response to the global action plan on AMR^2^ and in recognition of the increasing awareness of the important role that the environmental sector plays, a global quadripartite collaboration^4^ has been established among the World Health Organization (WHO), Food and Agriculture Organization (FAO), World Organisation for Animal Health (WOAH), and United Nations Environmental Programme (UNEP). At the European level (defined as European Union [EU] and European free trade association countries [EFTA] hereafter), surveillance of harmonised data and integrated reporting of antibiotic resistance in key zoonotic and indicator bacteria from humans, animals, and the food chain has been ongoing since 2010^5^ under the joint efforts of the European Food Safety Authority (EFSA) and European Centre for Disease Prevention and Control (ECDC). Although a shift towards integrating surveillance data from the human and food-producing animal sectors is more perceptible, inclusion of the environmental sector in the global One Health efforts remains underrepresented and is at its early stages.^6^^,^^7^

Surveillance systems can collect different microbiological, clinical, and demographic data, which should be reported to relevant stakeholders in a structured and meaningful manner in order to efficiently and consistently drive actions to mitigate AMR. Recent surveillance protocols and guidance documents from major authorities and stakeholders extensively support the harmonized collection and analysis of AMR and antimicrobial consumption (AMC) surveillance data in the human and animal sectors at local, national, and international levels.^8^^,^^9^ With the recognition of the importance of a One Health approach that also includes the environmental sector by the AMR community, the need for guidance on interlinking data from all the three sectors has come to the fore.

The COMBACTE-MAGNET EPI-Net network, a European public-private partnership project, has been working towards harmonising AMR surveillance strategies and developing tools for improving surveillance since 2015.^10^ In 2018, the COACH (Consensus group on antimicrobial surveillance to drive stewardship) project^11^ was launched with the aim to systematically summarise evidence on how to report AMR surveillance data to inform antimicrobial stewardship (AMS) teams in healthcare settings. The review did not identify any document providing indications on if and how AMR data from the animal sector from regional/national surveillance systems should be included to inform AMS in the human sector. A subsequent updated literature review and consensus exercise carried out in collaboration with the JPIAMR ARCH Network^12^, ^13^, ^14^, ^15^ further highlighted the need for recommendations that are inclusive of the environmental sector and antimicrobial residues surveillance.

Using the lessons learnt from the previous efforts and to support the ongoing global initiatives, a consensus project was launched by EPI-Net in March 2021 to identify the most important actions for the human, animal, and environmental sectors toward implementing a One Health approach. Given the complex epidemiological interconnectedness and heterogeneous surveillance approaches at play, the task of combining multisectoral data from routine AMR and AMC/antimicrobial residue surveillance is a challenging endeavour that requires targeted guidance. The main objective of this initiative was to bring together a multidisciplinary panel of experts from all three sectors and discuss how to combine and report full-scale AMR and AMC/antimicrobial residues surveillance data from the One Health triad in order to drive judicious antibiotic usage in the human sector.

## Search strategy and selection criteria

For this objective we utilised a modified Delphi approach. An expert panel was selected and an evidence-based consensus building exercise was carried out between March 2021 and January 2022.

### Expert panel selection

To identify potential experts for the panel, we reviewed: 1) recent publications on One Health surveillance; 2) ongoing European One Health consortia or projects website; 3) experts from the COACH project.^11^ A preliminary list of experts was created and an invitation for participation in the EPI-Net One Health consensus project was sent to 95, 58% of whom (55 experts) responded positively. These experts received a preliminary project protocol for perusal; a response with a feedback was considered as a further confirmation for participation in the initiative. Following substitutions in case of unavailability or additional referrals, a panel of 56 experts and stakeholders from 20 countries (52 high income, 4 upper middle or lower income) with expertise and active contribution to mitigation of the global issue of AMR and AMC, encompassing the three target sectors, and representing diverse backgrounds including academia and industry, constituted the EPI-Net One Health consensus working group (see Supplementary Table S1 for detailed information on the panel).

### Consensus building

To formulate the consensus statements which would form the backbone of the panel's recommendations, a multi-step approach was used: 1) development of key questions for the Delphi process; 2) literature review to summarise evidence; 3) presentation of the questions and evidence to the expert panel to obtain their preliminary statements via an online survey; 4) collation of statements; 5) first round of consensus via an online questionnaire; 6) second and final round of consensus during a virtual meeting on 3 December 2021; and 7) revisions, finalization, and approval of statements by the expert panel. Briefly, a preliminary set of questions was identified *a priori* by the research team and refined by the expert panel. Alongside this, a two-pronged literature search was carried out to review: a) recommendations available to support One Health reporting of AMR and AMC surveillance data; and b) current practices in reporting One Health surveillance data. Pertinent literature, i.e., guidance documents and One Health surveillance reports published between 2012 and 2021 in English Language were identified. A detailed description of the search and data extraction strategy is available on the EPI-Net website^16^ and Supplementary Appendix 1. Subsequently, the panel was presented with the questions and evidence summary in an online survey and requested to provide their expert opinions in the form of preliminary statements. The various responses received for each question was pooled, sorted by similarities, and transformed into comprehensive statements and incorporated in an online questionnaire^16^ on which the panel was asked to cast its votes measured on a 9-point Likert scale (range 1–9, with 1 indicating strong disagreement and 9 strong agreement). Consensus was considered to be achieved when the median score was ≥8 with at least 70% of the experts scoring in the highest tertile. The results of the survey were then presented at an online virtual meeting held on 3 December 2021. Questions not achieving consensus from the previous round were discussed in detail to drive the final consensus. After the meeting, the statements were reviewed, revised, and approved by the expert panel.

## Overall outcomes

### Delphi questions

In all, 16 key questions were identified in order to support structuring and reporting of full-scale multisectoral. These questions concerned three major aspects in the thematic of AMR and AMC/antimicrobial residues reporting from the human, animal, environmental sectors:

1. Common definitions and strategies: What should be the frequency and language of reporting? Should a One Health surveillance report replace the individual sector-specific surveillance reports? Should data from both public and private (industry-funded) sectors be included? In addition, for the evolving concept of environmental surveillance, what should be the common definition for the term “environment” to ensure homogenous data reporting?
2. Data reporting from AMC/antimicrobial residues surveillance: Should data on AMC and antimicrobial residues from all three sectors be reported in a One Health surveillance report? If so, data for which antimicrobials should be included and what should constitute data for the purpose of reporting? Should the results of a comparative or integrated analysis be additionally reported, and in this case how should the analysis be performed? Should information on cases of falsified and substandard antimicrobials be reported, and why?
3. Data reporting from AMR surveillance: Should data on AMR from all three sectors be reported in a One Health surveillance report? If so, data for which bacteria–drug combinations should be included and what should constitute data for the purpose of reporting? Should the results of a comparative or integrated analysis be additionally reported, and in this case how should the analysis be performed?

### Evidence

Twelve guidance documents were identified in total (see Supplementary Table S2). Among these, the ‘WHO integrated global surveillance on ESBL-producing *Escherichia coli* using a “One Health” approach’ provides the most comprehensive guidance for surveillance and reporting of AMR from the One Health triad.^17^ After screening 52 AMR and AMR/AMC surveillance reports published periodically in Europe, USA, Canada, Australia, New Zealand, and Japan, 18 (35%) One Health reports were found (see Supplementary Table S3) of which only 7 reported data from environmental sampling. The search indicated that no specific documents or recommendations were available to provide indications on combining full-scale AMR and AMC/AR surveillance data from all the three sectors.

### Consensus

The Delphi questions, together with summary documents^16^ generated from the literature and a glossary of definitions for the use of the terms across the project (Supplementary Table S4), were used to draft 35 initial statements that were surveyed for consensus in the next step. For 12 statements, consensus was achieved in the first round, while four questions that did not reach consensus were discussed in detail during the virtual meeting for a second round of consensus. An outline of the expert panel recommendations is provided in Table 1. Within the ensuing text we elaborate these statements to elucidate the rationale.

**Table 1:** Outline of EPI-Net One Health consensus working group recommendations.

Theme	Subtheme	Integrated actions
One Health approach for common definitions and strategies within surveillance reporting	Definition of environment	Environment should be considered as “the combination of physical, chemical, and biotic factors (e.g. climate, soil, and living beings) that act upon an organism or an ecological community and have a role in its form and survival, and which are not yet covered by human and animal surveillance efforts”^a^ (See Box 1 for full definition)
	Frequency of reporting	A One Health surveillance report should be published in addition to sector-specific unilateral reports. Surveillance data should be reported yearly. Time between collection of data and data access should be within 1–2 years. Reporting every two years could be considered in case of limited infrastructure and resources. In case of an emerging, serious pattern of resistance (i.e., with clinical impact for human health) shorter reporting times should be considered.
	Language of reporting	English should be prioritised, while local languages can be used for summaries.
	Inclusion of private (industry-funded) sector surveillance data	Industry-generated antimicrobial resistance (AMR), antimicrobial consumption (AMC), antimicrobial residues surveillance data should be included when available in a separate section within a One Health surveillance report. Anonymity should be guaranteed and quality assessment of the methodology is strongly advised.
One Health approach for antimicrobial consumption and residue surveillance reporting	Inclusion of AMC and antimicrobial residues data	AMC and antimicrobial residues data from the human, animal, and environmental sectors should be included in a One Health surveillance report. AMC data should be provided regularly, while concentration of antimicrobial residues data can be provided whenever available.
	Target antimicrobials for reporting	It would be preferable to identify a set of antimicrobials, such as A) the most commonly used antimicrobials in humans (including the critically important, the highly important and the important antibiotics defined by the World Health Organization),^18^ B) those related with recent rapidly emerging AMR of human clinical relevance over the last 5 years, and C) Antimicrobial Advice Ad Hoc Expert Group categorisation antimicrobials^19^ for data reporting in a One Health surveillance report.
	Inclusion of information on falsified and substandard antimicrobials	When available information on falsified and substandard antimicrobials should be included in a One Health surveillance report. Its inclusion need not be prioritised, but qualitative data could be included in a separate section within a One Health surveillance report.
	Core elements for data reporting	AMC and antimicrobial residues data reporting within a One Health surveillance report should be based on a standard set of metadata. The type and level of reporting is dependent on data availability and the sector.^b^ (see Table 2 for a detailed list of core elements by sector)
	Metrics	AMC data for target antimicrobials should be reported as defined daily doses (DDD) for the human sector; mg per kg and DDD for for the animal sector; mass per mass or volume for the environmental sector.^b^ (see Table 2 for elaborated metrics by sector)
	Integrated/comparative analysis	All raw data from the three sectors should be presented separately. Standard analysis by sector should be performed first and an integrated analysis can be performed additionally whenever possible. When common metrics cannot be identified, thus making it difficult to perform an integrated analysis, the description of trends by using the same metric for a given sector over time can be done to allow comparisons.
One Health approach for antimicrobial resistance surveillance reporting	Inclusion of AMR data	AMR surveillance data should be reported in a One Health report including results from the human, animal, and environmental sectors. Depending on the origin of the AMR data (the bacteria/samples), this will be the most comparable data between the three sectors.
	Target bacteria–drug combination for reporting	A One Health Report should include data on a set of bacteria–drug combination of common interest for the three sectors, and all additional pathogens data can be published as supplementary material or in unilateral reports.
	Core elements for data reporting	As with the AMC and antimicrobial residues data, AMR surveillance data reporting within a One Health surveillance report should also follow a standard set of metadata based on data availability and sector.^b^ (see Table 2 for a detailed list of core elements by sector)
	Metrics	Percentage of resistance with the denominator total isolates tested should be reported for target bacteria–drug combination in each sector, and when available data can be supplemented with AMR proportions by phenotypic (e.g. ESBL/AmpC) or genotypic (e.g. CTX-M) profiles of interest.^b^ (see Table 2 for elaborated metrics by sector)
	Integrated/comparative analysis	Overall AMR surveillance data should be presented by each sector separately. An additional comparative or integrated analysis is recommended and should be performed whenever possible, always accounting for (likely) biases. When the data are robust and experts are available, statistical analysis from simple correlation and regression analyses to more complex modelling exercises can be included.

a For a detailed definition of the term environment, see Box 1.

b The core elements and metrics were defined in detail by the expert panel and for easy readability, these are provided separately in Table 2.

## Integrated actions—A proposal from the EPI-Net One Health consensus working group

### Scope

The proposals from the expert panel serve as guidance to any stakeholder aiming to disseminate or communicate One Health surveillance data to have an impact at the local, national, or global level. This consensus is especially addressed to individual or organizational leaders in human, animal, and environmental health with an impact on the practices and policies in all three sectors. It also serves to optimise surveillance efforts and reporting. This is a first attempt at combining full-scale One Health triad data with the purpose of a better holistic understanding of global health. The provided consensus is mostly designed based on settings with high income where abundant resources allow the implication of all the contributors to human, animal, and environmental health. Although applying this guidance at global level would be desirable, implementation in low- and middle-income countries (LMICs) could be challenging and should be further explored in dedicated guidance documents.

### One Health approach for common definitions and strategies within surveillance reporting

#### Definition of environment

Although there have been advances in the monitoring of AMR and AMC/antimicrobial residues at the human-animal-environment interface (Supplementary Table S3^20^^,^^21^), a heterogeneity in the definition of “environment” is yet to be concretely addressed (Supplementary Table S5). Given that the framework for surveillance of AMR, AMC, and antimicrobial residues in the environment is still in its infancy and much is to be learned, many aspects of surveillance in this sector require further elaboration; however, without a common definition for what constitutes an environment for the purpose of monitoring AMR, AMC, and antimicrobial residues, reporting relevant and comparable data cannot be achieved. Based on this and taking the existing definitions into consideration, the term “environment” was discussed and defined in detail by the expert panel and is highlighted in Box 1.

Box 1 **Definition of environment for One Health AMR and AMC/antimicrobial residues surveillance data reporting.**

Environment is “the combination of physical, chemical, and biotic factors (e.g. climate, soil, and living beings) that act upon an organism or an ecological community and have a role in its form and survival, and which are not yet covered by human and animal surveillance efforts.” Within this context, the term "environmental sector" should comprise:

▪ Emission points
  o Wastewater, including wastewater from the community and hospital settings, pharmaceutical companies and wastewater treatment plants, maritime shipping (ballast water)
  o Farm effluents
  o Aquaculture residual water
  o Soil (e.g. contaminated by effluents or wastewater)
  o Air (e.g. contaminated by effluents or wastewater)
▪ Exposure points
  o Drinking water
  o Surface water (e.g. recreational water)
  o Food of plant origin (e.g. raw vegetables)
  o Wildlife
On a larger perspective, human and animal behaviours, as well as social and cultural factors, can also be considered “environment”; however, for the time being such definitions would probably not lead to actionable surveillance measures and ultimately would not serve the purpose of One Health surveillance.

#### Reporting frequency and language

As an integrated approach to AMR, AMC, and antimicrobial residues surveillance becomes common, the relevance of sector-specific reports was discussed with the expert panel. There was agreement that the significance of sector-specific reports cannot be overlooked. Since the three sectors are diverse, the panel recommended that a One Health surveillance report encompassing the most common and relevant aspects of the three sectors should be produced in addition to unilateral, sector-specific reports. The frequency of reporting was however extensively debated. Providing timely access to surveillance data is key; however, when both sector-specific and One Health surveillance reports are to be produced, feasibility plays a crucial role. Although agreement was achieved that the production of a yearly report would be ideal, the panel recognised that yearly reporting could result in excessive workload. Therefore, it was suggested that reporting of surveillance data every 2 years could be considered in case of limited infrastructure and resources. In case of the emergence of a serious resistance pattern that could have a clinical impact for human health, shorter reporting times could be considered to facilitate immediate response. The panel also agreed that for a One Health report a common language (English) should be used and prioritised to enable comparability, while local languages could be used to summarise only key information.

#### Private sector data inclusion

The need to share surveillance data between the public and private sectors at both the local and global levels is recognised as a clear need.^22^ Pharmaceutical companies generate a vast amount of information on AMC and AMR, which is only partially (if at all) available to the public. In 2017, the AMR Industry Alliance was established and a number of pharmaceutical companies signed the AMR Industry Declaration^23^ and Industry Roadmap for Progress on Combating Antimicrobial Resistance,^24^ which includes an industry commitment to share AMR surveillance data. More recently, four key actions were identified and proposed by Wellcome,^22^ including the need to “enable open innovation and data sharing within the AMR community” and “to facilitate the development of common methodological and metadata standards and data governance frameworks to enable data use by the scientific and public health community and allow data comparison with existing in-country datasets”. The Wellcome Trust-Open Data Institute pilot project which helped define these key actions has resulted in the establishment of the ‘Vivli AMR register’—a single platform for industry to share their surveillance data and provide coordinated access.^25^ In alignment with these recent developments, the expert panel agreed that the inclusion of AMC and AMR surveillance data from the private sector within a One Health report will indeed increase the usefulness of these data. However, information such as the source of data, sampling strategies, antibiotic susceptibility testing methodology, and other details including funding should be differentiated among different pharmaceutical companies. Therefore, the inclusion of (anonymised) industry data was suggested to be implemented in a separate section together with a description of data collection methods.

### One Health approach for antimicrobial consumption and residue surveillance reporting

#### AMC and antimicrobial residues data inclusion

To date, there are no well-established guidelines for surveying antimicrobial residues in the environmental sector, and a common strategy for the collection and reporting of AMC surveillance data and antimicrobial residue data encompassing the human, animal, and environmental sectors has not yet been fully developed. Within a One Health report, provision of reliable and comparable AMC surveillance data is essential to understand the epidemiology of AMR and to identify areas for potential intervention. Consequently, the panel agreed that AMC data are necessary and should thus be regularly provided. Although knowledge of environmental concentrations is critical to understand the risk of environmental selection, data on antibiotic residues are usually not reported (Supplementary Table S3). The panel agreed that if antimicrobial residues or concentration data are available, they should be reported; if they are not available, minimum AMC data from the human and animal sectors should be provided within the report.

#### Target antimicrobials

In 2005, the WHO proposed categorisation of antibiotics classified as Critically Important Antimicrobials (WHO CIA) for human health, which is periodically revised.^18^ In 2019 the ‘WHO AWaRe classification of antibiotics for evaluation and monitoring of use’ was proposed, identifying three categories of antibiotics (“Access”, “Watch” and “Reserve”) to support access ensuring at the same time good stewardship.^26^ Efforts to harmonise CIA and AWaRe lists are currently ongoing, and in 2020, the Antimicrobial Advice Ad Hoc Expert Group (AMEG) expanded the WHO CIA list to establish four categories of antibiotics based on the potential consequences of increased antimicrobial resistance for public health when used in animals.^19^ More recently the WOAH's ‘OIE List of Antimicrobial Agents of Veterinary Importance’ has also been published.^27^ Considering all these ongoing efforts, the panel set out to define a set of antimicrobials for which AMC data should be reported within a One Health report and concluded that high priority should be given to the most commonly used antibiotics in the human sector, including those defined as critically important, highly important, and important by the WHO CIA list, and to those related with rapid emergence of AMR and clinical relevance in the human sector.

#### Substandard and falsified (SF) antimicrobials

Since substandard and falsified (SF) antimicrobials drive the emergence and development of AMR, and antimicrobials are among the most commonly reported SF medical products worldwide,^28^ the inclusion of information on reported cases of SF was discussed among experts. Due to their impact on the selective pressure of AMR and on the mitigation measures that can be adopted by prescribers, the expert panel concluded that their inclusion as an addendum to a One Health report should be considered. Although a medicine quality surveillance network already exists at the global level,^28^ the experts suggested to include this information in the One Health report. The inclusion of descriptive (qualitative) data on reported SF antimicrobials could not only bring attention to the actual issue and underpin policy actions, but also highlight the importance of considering them in data analysis and interpreting trends in AMR and AMC.

#### Core elements and metrics

Clear definition and standardisation of metrics allows comparative approaches. Many of the routinely used surveillance indicators for AMC are highly sector-specific and the panel recognised the need for a trans-sectorial overarching reach to ensure comparability and compatibility of data. A list of core elements and metrics identified by the expert panel for AMC data reporting is shown in Table 2. It should be noted that all metadata recommended by current guidance documents (Supplementary Table S2) and those reported by current One Health surveillance reports (Supplementary Table S3) guided the definition of core elements and metrics. In terms of the environmental sector AMC is not a collectable data source, since there is always spill-over from the other sectors (animal, human). The equivalent is therefore the collection of antimicrobial residues/concentrations measured in mass per mass or per volume depending on liquid (e.g. water) sources or solid sources. Since sampling type and measures are very important in this sector and often differing these are the most important metadata that need to be reported. For the human and animal sectors, additional considerations such as route of administration, population (healthcare versus community for human; target animals for animal sector), and anatomical therapeutic chemical codes were recommended as core elements by the panel. As for the metrics, defined daily doses (DDD) with setting-specific denominators was proposed for the human sector. Duration of therapy (DOT) and DDDs per package (PID) was suggested to be reported when available.

**Table 2:** Surveillance metrics and core elements for One Health AMR and AMC/antimicrobial residues surveillance data reporting.

		Core elements	Metrics
Human sector	AMC	Sampling type Anatomical Therapeutic Chemical (ATC) code Sales or prescriptions Metrics Route of administration Setting (healthcare vs community)	**Minimum:** defined daily doses (DDD) per 1000 inhabitants per day (community setting) and DDD per 1000 patient days (hospital setting). **Additional**: duration of therapy (DOT), DDDs per package (PID).
	AMR	Surveillance population/setting/sub-setting Surveillance type: mandatory vs. voluntary Bacteria-drug combination Status i.e. infection vs colonization Specimen Demographics Domestic vs travel-related cases Infection type Place of acquisition - community vs healthcare vs hospital Denominator data Susceptibility testing methods Quantitative (zone diameter or MIC) and qualitative (S, I, R) susceptibility test results Additional and relevant test results Resistance interpretation guidelines Resistance genes	**Minimum**: Percentage of resistance with the denominator of total isolates tested (should be de-duplicated, so that one isolate represents one patient). When available supplemented with: o Proportion of AMR phenotypic profiles of interest for each sector (e.g. ESBL/AmpC) o Proportion of AMR genes of interest for each sector (e.g. CTX-M) o Proportion of clinically and epidemiologically (e.g., non-wild type) 'resistant' isolates to allow for more detailed assessments over time/different locations **Additional**: Population-based incidence/prevalence rates
**Animal sector**	AMC	Target animals (stratification by animal species) Sampling type Anatomical Therapeutic Chemical in animal medicine (ATCvet) code Sales or prescriptions Metrics Route of administration	**Minimum**: mg per kg of estimated biomass, and defined daily dose for animals (DDDvet) per kg of biomass.
	AMR	Target animals Setting/sub-setting Surveillance type: mandatory vs. voluntary Bacteria-drug combination Sampling type (diseased and infection type, vs healthy) and domestic vs imported Specimen Denominator data Susceptibility testing methods Quantitative (zone diameter or MIC) and qualitative (S, I, R) susceptibility test results Additional and relevant test results Resistance interpretation guidelines Resistance genes	**Minimum**: Percentage of resistance with the denominator of total isolates tested. When available supplemented with: o Proportion of AMR phenotypic profiles of interest for each sector (e.g. ESBL/AmpC) o Proportion of AMR genes of interest for each sector (e.g. CTX-M) o Proportion of clinically and epidemiologically (e.g., non-wild type) 'resistant' isolates to allow for more detailed assessments over time/different locations
**Environmental sector**	Antimicrobial residues/concentrations	Residues or micropollutant measurements Sampling type Metrics	**Minimum**: Mass per mass for solids (e.g. μg/kg) or mass per volume for liquids (e.g. μg/L).
	AMR	Setting Specimen Bacteria-drug combination Denominator data Susceptibility, susceptibility testing methods Quantitative and qualitative (S, I, R) susceptibility test results Resistance interpretation guidelines Resistance genes	**Minimum**: Percentage of resistance with the denominator of total isolates tested. When available supplemented with: o Proportion of AMR phenotypic profiles of interest for each sector (e.g. ESBL/AmpC) o Proportion of AMR genes of interest for each sector (e.g. CTX-M) o Proportion of clinically and epidemiologically (e.g., non-wild type) 'resistant' isolates to allow for more detailed assessments over time/different locations **Additional**: AMR gene abundance

For the animal sectors, mg of active substance per kg of estimated biomass and defined daily doses for animals (DDDvet) per kg of biomass should be used.^29^^,^^30^ Although there are some limitations in the use of this metric (i.e., a list DDDvet is not available for all species), it was concluded that it should be included if available.

#### Integrated/comparative analysis

Through the longstanding collaboration between the ECDC, EFSA, and EMA, inter-agency reports on integrated analyses of AMC and occurrence of AMR in bacteria from humans and food-producing animals (JIACRA) were published.^31^, ^32^, ^33^ These works offered an integrated analysis of the relationships between AMC in both the human and animal sectors and the occurrence of AMR in bacteria from the human and food-producing animal sectors using routinely gathered surveillance data.

The extension of such an approach to the environmental sector requires acknowledging the differences in the metrics currently used within and across the three sectors. Although the experts recognised that ongoing discussion is still needed to better understand how to utilise and analyse data across sectors, it was suggested that raw data from each sector should be presented independently within a report and that trends should be reported at a minimum. Integrated analyses across sectors could also be performed whenever possible. When common metrics cannot be identified, thus making it difficult to perform an integrated analysis, trends using the same metric for a given sector over time could be described to allow comparisons.

### One Health approach for antimicrobial resistance surveillance reporting

#### AMR data inclusion

When appropriate sampling and bacteria–drug combinations are targeted, data generated through AMR surveillance activities from the human, animal, and environmental sectors can provide the most comparable evidence necessary to understand the complex epidemiology of AMR and establish policy and research actions.^34^ Moreover, it is widely accepted that AMR surveillance data are essential to guide AMS activities in the human sector.^35^ Therefore, linking multisectoral AMR surveillance data for reporting was emphasised by the expert panel as being central to the evolving national and global agenda against AMR in accordance with observations from global organisations.

#### Target bacteria–drug combination

In 2017, the WHO prioritised for the first time a list of antibiotic-resistant bacteria to guide global research and discovery of new antibiotics^36^^,^^37^ for the human sector. The WHO priority pathogens list not only supports the development of new antibiotics, but also highlights key resistant pathogens that should be monitored for effective infection control and antibiotic policies. Furthermore, the recommendations of the WHO Advisory Group on Integrated Surveillance of Antimicrobial Resistance (AGISAR) provide a substantial outset for the definition of bacteria–drug combinations of common interest in the human and animal sectors.^38^ More recently, the tricycle protocol for integrated surveillance in the human, animal, and environmental sectors has identified extended-spectrum beta-lactamase producing *E. coli* as an indicator organism.^17^ However, there are currently no overarching guidelines available to support the identification of bacteria–drug combinations considering the inclusion of surveillance data from all three sectors, thereby highlighting a need for prioritisation and the creation of a global priority list for One Health surveillance and reporting. In this context, the expert panel recommended that AMR surveillance data in a One Health report should mainly focus on a set of resistant bacteria of common interest to the three sectors with specific attention to transmissibility of resistance across sectors and infectivity, while all additional data on sector-specific bacteria can be published as Supplementary Material or in unilateral reports for parties that are interested in accessing the full dataset relevant to each sector.

#### Core elements and metrics

To support the interpretation of the AMR estimates, values must be accompanied by background information collected as part of a surveillance system. The concept of surveillance of AMR is well established, but the quality of data generated highly depends on standardised reporting and collection and often varies across sectors. The expert panel, therefore, defined core elements and associated metrics to provide a definition for data reporting from the different sectors (Table 2). Both combined form the basis for achieving standardised and cross-sectoral one health surveillance data reporting. Setting, specimen, bacteria–drug combination and specifics on microbiological investigation and interpretation were identified as being core elements in all sectors. This has critical importance in order to allow for meaningful conclusions in the medium- and long-term. Furthermore, status of health or illness (i.e. infection or colonisation) and source (i.e. domestic or travel-related/imported) were identified as additional core elements for the human and animal sectors. Although some priority is suggested for specific sectors and settings—in the animal sector, for example, samples from healthy animals can provide an unbiased measure of AMR in source for the human food supply—the panel agreed that all aspects should be addressed, whenever possible. It is acknowledged that data might not be available for all the core elements. Nevertheless, the panel agreed that reporting these elements should nonetheless be encouraged and strived for.

Furthermore, the ability to test AMR in various sectors is very different. For example, molecular diagnostics of AMR using solely culture-independent techniques (CITs) is not a norm for surveillance in the human and animal sectors. Due to the necessity of culture-based phenotypic antibiotic susceptibility testing methods in detecting clinically-relevant levels of antibiotic resistance, low cost/complexity molecular techniques typically complement phenotypic methods of AMR detection.^39^^,^^40^ In the environmental sector, on the other hand, the approach to AMR diagnostics takes a different strategy due to challenges that cultivation of bacteria from environmental samples pose and the potential objective of surveillance being transmission and evolution of resistance.^41^ In the present scenario of bacterial culture-dependent, isolate-based surveillance, the expert panel recommended the reporting of AMR as the proportion of resistant isolates to total isolates tested in each sector. When available, this estimate can be supplemented with the proportion of specific genotypic/phenotypic/epidemiological profiles among the isolates tested (Table 2). Furthermore, inclusion of population-based metrics for the human sector should be considered whenever available. With the role of CITs being more profound in the environmental sector, inclusion of absolute numbers generated by CITs was recommended for this sector.

#### Integrated/comparative analysis

Evidence on the role of ‘spill-over’ has been well established for AMR; although spill-over points have been mainly evaluated in the community and hospital settings (wastewater/sewage), progress in other settings is currently being made. The expert panel therefore agreed that even though spill-over points are largely under research and integrated analyses are aspirational, they can still be performed whenever feasible to give an overview of how the different sectors influence each other, where potential interventions can be deployed, and help identify research hypotheses. Thus, it was recommended that the inclusion of results from additional comparative or integrated analysis, always accounting for (likely) biases, should be considered for added value. References to current One Health surveillance reports that include a combined analysis are provided in Supplementary Table S3.

### Limitations

The recommendations have some limitations. Firstly, the panel mainly assessed antibiotics although role of other substances such as biocides should be included. Secondly, some recommendations may lack specificity due to lack of internationally-recognised gold standard. Finally, transferability could be limited to economic resources of countries. These limitations and suggestions for future research are detailed in Box 2.

Box 2 **Summary of limitations and grey areas for future research.**

**Table:** 

Limitation	Consideration for future research
• The EPI-Net One Health consensus recommendations were developed with the intention to support reporting of One Health surveillance data to impact antibiotic policies in the human sector and limit the spread of antimicrobial resistance (AMR) in human healthcare. Focus on antimicrobials was limited to antibiotics, and the role of biocides was not addressed.	• Future One Health initiatives should aim to comprehensively approach the problem of AMR including biocides as antimicrobials and evaluating FAIR (Findable, Accessible, Interoperable, and Reusable)^42^ data and open-source data repositories for One Health surveillance data.
• Some recommendations lack specificity (for example, on the methods for an integrated analysis of data from the three sectors) as there are currently no established, gold-standard methods. Prioritisation of target pathogens and antibiotics for monitoring, elaboration of data collection, specification of microbiological methods, and description of epidemiological variables were not addressed.	• Surveillance of AMR and antimicrobial residues in the environmental sector is still in the initial phases and much is to be learned and defined. The recommendations issued by the EPI-Net One Health consensus group were based on the current epidemiological situation. One Health recommendations for reporting should be constantly adjusted based on changing epidemiological scenario. Prioritization exercises are needed to clarify indicators for both surveillance and data reporting within a One Health perspective.
• Even though feasibility was given utmost importance and recommendations were balanced with minimum/additional options, an exhaustive delineation of the recommendations by economic setting and regional differences in AMR could not be addressed. Participation of experts from non-European countries was limited although specific consideration on transferability and generalisability of recommendation has been considered throughout.	• One Health surveillance and reporting strategies should be tailored to low- and middle-income countries, where economic and personnel resources may be limited.
	• A validation exercise where the EPI-Net One Health consensus group's recommendations are put in place, involving the three sectors in different countries, would highlight the preparedness to apply such recommendations, gaps that need to be addressed and that eventually require the engagement of national regulatory organisations to make them more actionable.

## Conclusions

The recommendations from the EPI-Net One Health consensus working group represent the first practical outline to support multisectoral data reporting of antimicrobials resistance rates and usage. We believe the document can support discussion among major international and national stakeholders for coordinated One Health surveillance reporting from the human, animal, and environmental sectors within national and regional plans for antibiotic policy to reduce the burden of AMR.

## Contributors

Contributors’ initials listed alphabetically when multiple authors were involved in the same task. ET conceived the consensus initiative. NBR oversaw the conduct of the project. CMB, FA, LG, MC, MT, and NBR drafted the protocol. CMB, FA, LG, MT, and NBR carried out the finalization of the protocol; literature search; data extraction; multiple survey organization and results analysis; organization of the virtual consensus meeting and collation of the resulting discussions; finalization of statements. EM, ET, GDA, JRB, MDP, NM, and OS chaired the virtual consensus meeting. CMB wrote the preliminary draft of the manuscript; FA and NBR revised it to develop the first draft of the manuscript; all co-authors contributed to review and feedback of this first draft. NBR finalized the manuscript together with CMB, ET, FA, GDA, LC, MDP, NM, OS, RD, and SK. All co-authors approved the final version. ET was responsible for the final decision to submit the manuscript.

## Declaration of interests

JA received grants/contracts from the Spanish Ministry of Agriculture, Food and Fisheries and EU Horizon 2020. Work of BPG was funded by the German Center for Infection research Clinical Research Unit (DZIF-CRU) at Tübingen. RD participated on Data Safety Monitoring Board of the ASTARTÉ study (for which no payments were received). WG received grants/contracts from the Horizon Europe grant (supported by UKRI) on AMR and pathogen evolution in costal environments and the UK Natural Environment Research Council grants on AMR Knowledge Exchange NE/V019279/1 and AMR evolution NE/W006251/1; WG received consultation fee for EU DG Sante AMR policy evaluation and recommendations. Work of AK was funded in the context of the project One Health EJP, which has received funding from the European Union's Horizon 2020 research and innovation programme under Grant Agreement No. 773830. EP received grants/contracts from the Austrian Ministry of Health for the National Surveillance Network of healthcare-associated infections (ANISS); EP received honorarium for participation as a chair of the Advisory board on AMR and MDRO Pfizer Austria (27.09.22). MB received grants/contracts from Janssen Vaccines, Novartis, CureVac, and Merck; MB received payment or honoraria for lectures, presentations, speakers bureaus, manuscript writing or educational events from Takeda (November 2019); MB participated on Data Safety Monitoring Board or Advisory Board of Sanofi, Spherecydes, Pfizer, Merck, Novartis, and Astra-Zeneca. MM received support for attending meetings and/or travel from the Global Antibiotic Research and Development Partnership (12.10.22–13.10.2022). LS received grants or contracts from the JPIAMR network grant 2020; LS received support for attending meetings and/or travel from ESCMID for the attendance of ECCMID 2022. DT is an employee of GlaxoSmithKline and holds shares in GlaxoSmithKline. TVB received consultation fees from Stonehaven Consulting; TVB received payment or honoraria for lectures, presentations, speakers bureaus, manuscript writing or educational events from Swedish Veterinary Council. Work of AV was supported by the COMBACTE-MAGNET consortium; AV serves as the ISAC president (International Society of Antimicrobial Chemotherapy) and board member of NVMM (Dutch Microbiology Society). All other authors declare no competing interests.
