# Supplementary material for: EPI-Net One Health reporting guideline for antimicrobial consumption and resistance surveillance data: a Delphi approach
Source: Lancet Reg Health Eur. 2022 Dec 22;26:100563. doi: 10.1016/j.lanepe.2022.100563 (PMC9989632; doi:10.1016/j.lanepe.2022.100563)
Supplement: Supplementary Material [file mmc1.docx]

**Supplementary Material**

**Authors**: author name(s), preferred degree (one only).

Nithya Babu Rajendran, PhD†, Fabiana Arieti, PhD†, Carla Alejandra Mena-Benítez, MSc†, Liliana Galia, PhD‡, Maela Tebon, PhD‡, Julio Alvarez, PhD, Beryl Primrose Gladstone, PhD, Lucie Collineau, PhD, Giulia De Angelis, PhD, Raquel Duro, MD, Prof. William Gaze, PhD, Siri Göpel, MD, Prof. Souha S. Kanj, MD, Prof. Annemarie Käsbohrer, DVM, Direk Limmathurotsakul, PhD, Estibaliz Lopez de Abechuco, PhD, Elena Mazzolini, MSc, Prof. Nico T. Mutters, MPH, Maria Diletta Pezzani, MD, Prof. Elisabeth Presterl, MD, Hanna Renk, MD, Prof. Jesús Rodríguez-Baño, PhD, Oana Săndulescu, PhD, Federico Scali, PhD, Robert Skov, MD, Prof. Thirumalaisamy P. Velavan, PhD, Cuong Vuong, PhD and Prof. Evelina Tacconelli, PhD* on behalf of the EPI-Net One Health consensus working group¥

**¥** **Members of the One Health consensus working group**

Ayola Akim Adegnika, PhD, Lisa Avery, PhD, Prof. Marc Bonten, PhD, Alessandro Cassini, MD, Claire Chauvin, PhD, Monica Compri, MD, Peter Damborg, PhD, Sabine de Greeff, PhD, Maria Dolores Del Toro, PhD, Matthias Filter, MSc, Alison Franklin, PhD, Bruno Gonzalez-Zorn, PhD, Kari Grave, PhD, Prof. Didier Hocquet, PhD, Ludwig E. Hoelzle, DVM, Erta Kalanxhi, PhD, Ramanan Laxminarayan, PhD, Leonard Leibovici, MD, Surbhi Malhotra-Kumar, PhD, Prof. Marc Mendelson, PhD, Prof. Mical Paul, MD, Cristina Mu*ñ*oz Madero, DVM, Rita Murri, MD, Prof. Laura JV Piddock, PhD, Carolien Ruesen, PhD, Maurizio Sanguinetti, PhD, Thorben Schilling, MD, Remco Schrijver, DVM, Prof. Mitchell J. Schwaber, MD, Luigia Scudeller, MD, Didem Torumkuney, PhD, Thomas Van Boeckel, PhD, Wannes Vanderhaeghen, PhD, Prof. Andreas Voss, MD, Teresa Wozniak, PhD

†Equally contributing first authors.

‡Equally contributing second authors.

*Corresponding author.

**Table of Contents**

[Supplementary Appendix 1. Search and data extraction strategy 1](#_Toc117764387)

[Supplementary Table 1. List of experts involved. 3](#_Toc117764388)

[Supplementary Table 2. List of the guidance documents publicly available and published from 2016 to 2021 providing indications on how antimicrobial resistance and antimicrobial consumption/residues data should be included in a One Health report. 6](#_Toc117764389)

[Supplementary Table 3. List of One Health surveillance reports published at least once between 2018 and 2021 by publicly-funded national or international surveillance systems. 7](#_Toc117764390)

[Supplementary Table 4. Glossary of terms used across the text. 10](#_Toc117764391)

[Supplementary Table 5. Definition of environment in current guidance documents. 11](#_Toc117764392)

# Supplementary Appendix 1. Search and data extraction strategy

Data collection for evidence summary

In order to understand the most important elements that constituted an ideal One Health AMC/AMR surveillance report which combined data from humans, animals, and environment sectors, a multifaceted literature search and data collection was performed. The review of literature was divided in two main streams:

- Guidance documents and tools providing indications on how surveillance data on AMR and AMC should be included in a One Health report.
- Publicly available One Health surveillance reports providing examples on how data on AMR and AMC are currently reported in different countries.

Indications for One Health surveillance data reporting

A systematic search for guidance documents in English language published in the last 5 years, that provided indications on how AMR surveillance data should be reported, was carried out using Google search engine and a combination of the following terms: reporting AND antimicrobial resistance AND guidelines AND One Health AND environment. In addition, a review of guidelines already mapped and included in a previous consensus exercise (“Bridge the Gap: Survey to Treat” white paper series^1^) was performed to enhance the available literature resources. Any other guidance document suggested by the panel of experts was considered. Using the results of the 2019-2020 Tripartite AMR country self-assessment survey^2^ the available national action plans were identified and reviewed to detect available recommendations for reporting public health surveillance data. The information from the guidance documents was collected using predefined variables (Table S1a).

***Table S1a****: Variables guidance documents and tools*

| **Guidance documents** | | |
| --- | --- | --- |
| **Characteristics of the document** | **General content of the**  **document** | **Reporting** |
| Title  Document type  Agency or Journal  Year of publication  General purpose  Link or DOI | Sector: human, animal, environment  Compartments (within a sector)  Indications on dissemination and reporting  Definition of “environment” | Presence of indications for:  Antimicrobial consumption (AMC) sales and usage  Type of antimicrobial (ATC and ATCvet)  Metrics (tons, mg/kg biomass)  Overall and/stratified  Antimicrobial resistance (AMR)  Sample source (place)  Sample type (specimen)  Sample storage  Target bacteria  Target antimicrobials  Laboratory methods  Data management and analysis |

One Health surveillance reports: state-of-the art

Latest One Health surveillance reports published (between 2018 and 2021) by publicly funded national or international surveillance systems (human, animal and environment) were mapped to understand current reporting practices. The search was limited to pre-Brexit European Union (EU) and European free trade association countries (EFTA) countries, USA, Canada, Australia, New Zealand and Japan. European national surveillance reports were tracked using the freely-accessible EPI-Net central data repository (CDR)^3^, while extra-EU/EFTA national surveillance reports using the surveillance systems inventory from the EPI-Net AMR travel tool^3^, respectively.

Reports that included data on at least two sectors were collected. If a specific report for AMC and/or AMR in the environment was available, it was included as well. For the search of national environmental reports, a computerized literature search using Google search engine was conducted, using a combination of the following search terms in the local languages: ‘Antimicrobial resistance’, ‘Antibiotic resistance’, ‘Surveillance’, ‘Monitoring’, ‘Environment’, ‘Water’. Table S1b summarizes the variables used for data collection.

***Table S1b****: Variables national surveillance reports*

| **ONE HEALTH reports** | | |
| --- | --- | --- |
| **Characteristic of the report** | **General** | **Antimicrobial consumption (AM)** |
| Title  Country  Country income category  Acronym of surveillance system  Source: national *vs* international | Reporting language  Report publication frequency  Year of publication  Year of surveillance data  One Health report (3 sectors *vs* 2 sectors)  Environmental report (specific report tailored to environmental sector)  AMC and AMR results availability in the same report  Inclusion of industry surveillance data | Metadata  Target antimicrobials monitored  Metrics  AMC surveillance results stratification  Inclusion of integrated analysis or comparative analysis |
|  |  | **Antimicrobial resistance (AMR)** |
|  |  | Metadata  Target bacteria and drug combinations monitored (especially referred to common bacteria monitored in all sectors)  Indicators  AMR surveillance results stratification  Inclusion of integrated analysis or comparative analysis |

* Specific selective methods= methods based on selective plates

**Data synthesis**

The recommendations together with strengths and characteristics identified in the retrieved literature drove the development of:

1. A descriptive summary of evidence
2. Key Delphi questions
3. A set of core elements for One Health surveillance reporting to be discussed by the expert panel for consensus.

^1^ https://www.jpiamr.eu/projects/arch/

^2^ https://www.who.int/publications/m/item/tripartite-amr-country-self-assessment-survey-(tracss)-2019-2020

^3^ https://epi-net.eu/about/

# Supplementary Table 1. List of experts involved.

| **Degree** | **First Name** | **Last Name** | **Affiliation** | **Country** | **Country income Status*** |
| --- | --- | --- | --- | --- | --- |
| PhD. | Julio | Alvarez | VISAVET Health Surveillance Center and Department of Animal Health, Faculty of Veterinary Medicine, Complutense University, Madrid, Spain | Spain | HIC |
| PhD. | Lucie | Collineau | French Agency for Food, Environmental and Occupational health and safety, ANSES, Maisons-Alfort, France. | France | HIC |
| PhD. | Giulia | De Angelis | Dipartimento di Scienze Biotecnologiche di base, Cliniche Intensivologiche e Perioperatorie, Universita` Cattolica del Sacro Cuore, Rome, Italy | Italy | HIC |
| MD. | Raquel | Duro | Centro Hospitalar do Tâmega e Sousa, Penafiel, Porto, Portugal | Portugal | HIC |
| PhD. | William | Gaze | The European Centre for Environment and Human health, University of Exeter Medical School, University of Exeter, Penryn, Cornwall, UK. | United Kingdom | HIC |
| MD. | Siri | Göpel | Infectious Diseases, Department of Internal Medicine I, Tübingen University Hospital, Tubingen, Germany. German Centre for Infection Clinical Research Unit for healthcare associated infections, Tübingen, Germany (DZIF). | Germany | HIC |
| MD. | Souha | Kanj | Global Affairs, Division of Infectious Diseases, Infection Control Program, Antimicrobial Stewardship Program, American University of Beirut Medical Center, Beirut, Lebanon. | Lebanon | LMIC |
| DVM. | Annemarie | Käsbohrer | German Federal Institute for Risk Assessment, Berlin, Germany. | Germany | HIC |
| PhD. | Direk | Limmathurotsakul | Mahidol Oxford Tropical Medicine Research Unit and Department of Tropical Hygiene, Faculty of Tropical Medicine, Mahidol University, Thailand and Centre for Tropical Medicine and Global Health, Nuffield Department of Medicine, University of Oxford, United Kingdom | Thailand | UMIC |
| PhD. | Estibaliz | López de Abechuco Garrido | German Federal Institute for Risk Assessment (BfR), Department 4 - Biological Safety, Berlin, Germany | Germany | HIC |
| DVM. | Elena | Mazzolini | Department of Epidemiology, Istituto Zooprofilattico Sperimentale delle Venezie, Udine-Padova, Padua, Italy. | Italy | HIC |
| MPH. | Nico | Mutters | Institute for Hygiene and Public Health, University Hospital Bonn, Bonn, Germany. | Germany | HIC |
| MBA. | Elisabeth | Presterl | Department of Infection Control and Hospital Epidemiology, Medical University of Vienna, Vienna, Austria. | Austria | HIC |
| PhD. | Beryl Primrose | Glastone | Infectious Diseases, Department of Internal Medicine I, Tübingen University Hospital, Tubingen, Germany. German Centre for Infection Clinical Research Unit for healthcare associated infections, Tübingen, Germany (DZIF). | Germany | HIC |
| MD. | Hanna | Renk | University Children's Hospital Tübingen, Tübingen, Germany. | Germany | HIC |
| PhD. | Jesús | Rodríguez-Baño | Infectious Diseases and Microbiology Division, Hospital Universitario Virgen Macarena / Department of Medicine, University of Seville / Biomedicine Institute of Seville (IBiS), Seville, Spain, and CIBERINFEC, Instituto de Salud Carlos III, Madrid, Spain | Spain | HIC |
| PhD. | Oana | Sandulescu | Department of Infectious Diseases, Carol Davila University of Medicine and Pharmacy, Bucharest, Romania and. National Institute for Infectious Diseases "Prof. Dr. Matei Balș", Bucharest, Romania. | Romania | HIC |
| PhD. | Federico | Scali | Istituto Zooprofilattico Sperimentale della Lombardia e Dell'Emilia Romagna, Brescia, Italy. | Italy | HIC |
| MD. | Robert | Skov | Statens Serum Intitute | Denmark | HIC |
| PhD. | Thirumalaisamy P. | Velavan | Institute of Tropical Medicine, Universitätsklinikum Tübingen, Tübingen, Germany. Vietnamese - German Center for Medical Research, Hanoi, Vietnam. | Germany | HIC |
| PhD. | Cuong | Vuong | AiCuris Anti-infective Cures GmbH, Wuppertal, Germany. | Germany | HIC |
| PhD. | Evelina | Tacconelli | Infectious Disease Unit, Department of Diagnostic and Public Health, Azienda Ospedaliera Universitaria Integrata, Verona | Italy | HIC |
| PhD. | Ayola Akim | Adegnika | Institute of Tropical Medicine, University of Tübingen, and German Center for Infection Research (DZIF), Tübingen, Germany. Centre de Recherches Médicales de Lambaréné, Lambaréné, Gabon. | Gabon | UMIC |
| PhD. | Lisa | Avery | The James Hutton Institute, Craigiebuckler, Aberdeen, Scotland. | United Kingdom | HIC |
| PhD. | Marc | Bonten | Julius Center for Health Sciences and Primary Care, University Medical Center Utrecht, Utrecht University, Utrecht, The Netherlands. | The Netherlands | HIC |
| MD. | Alessandro | Cassini | Infection Prevention and Control Unit, Infectious Diseases Service, Lausanne University Hospital (CHUV), Lausanne, Switzerland. Deputy Cantonal Doctor, Public Health Department, Canton of Vaud, Lausanne, Switzerland. | Switzerland | HIC |
| PhD. | Claire | Chauvin | ANSES, Ploufragan-Plouzané-Niort Laboratory, Ploufragan, France | France | HIC |
| PhD. | Peter | Damborg | Department of Veterinary and Animal Sciences, University of Copenhagen, Frederiksberg, Denmark. | Denmark | HIC |
| PhD. | Sabine | De Greeff | Centre for Epidemiology and Surveillance of Infectious Diseases, National Institute for Public Health and the Environment, Bilthoven, the Netherlands. | The Netherlands | HIC |
| PhD. | Maria Dolores | Del Toro | Infectious Diseases and Microbiology Division, Hospital Universitario Virgen Macarena / Department of Medicine, University of Seville / Biomedicine Institute of Seville (IbiS), Seville, Spain. CIBERINFEC, Instituto de Salud Carlos III, Madrid, Spain. | Spain | HIC |
| MsC. | Matthias | Filter | German Federal Institute for Risk Assessment (BfR), Department 4 - Biological Safety, Berlin, Germany. | Germany | HIC |
| PhD. | Alison | Franklin | United States Environmental Protection Agency, US-EPA, Washington, D.C. United States of America. | United States of America | HIC |
| PhD. | Bruno | Gonzalez-Zorn | Antimicrobial Resistance Unit, Universidad Complutense de Madrid, Madrid, Spain. | Spain | HIC |
| PhD. | Kari | Grave | Department of epidemiology, Norwegian Veterinary Institute, Ås, Norway. | Norway | HIC |
| PhD. | Didier | Hocquet | Infection Control Unit, University Hospital of Besançon, France. | France | HIC |
| DVM. | Ludwig E. | Hoelzle | Institute of Animal Science, Department of Livestock Infectiology and Environmental Hygiene, Stuttgart, Germany. | Germany | HIC |
| PhD. | Erta | Kalanxhi | One Health Trust, Washington DC, USA. | United States of America | HIC |
| PhD. | Ramanan | Laxminarayan | One Health Trust, Washington DC, USA. | United States of America | HIC |
| MD. | Leonard | Leibovici | Faculty of Medicine, Tel-Aviv University, Tel-Aviv, Israel. Research Authority, Rabin Medical Center, Petah-Tiqva, Israel. | Israel | HIC |
| PhD. | Surbhi | Malhotra-Kumar | Laboratory of Medical Microbiology, Vaccine and Infectious Disease Institute, University of Antwerp, Antwerp, Belgium. | Belgium | HIC |
| PhD. | Marc | Mendelson | Division of Infectious Diseases and HIV Medicine, Department of Medicine, Groote Schuur Hospital, University of Cape Town, Cape Town, South Africa. | South Africa | UMIC |
| DVM. | Cristina | Muñoz Madero | Agencia Española del Medicamento y Productos Sanitarios (AEMPS), Coordinación del Plan Nacional Antibióticos (PRAN), Madrid, Spain. | Spain | HIC |
| MD. | Rita | Murri | Institute of Infectious Diseases, Fondazione Policlinico Universitario A. Gemelli IRCCS, Universitá Cattolica del Sacro Cuore, Rome, Italy. | Italy | HIC |
| MD. | Mical | Paul | Division of Infectious Diseases, Rambam Health Care Campus and The Ruth and Bruce Rappaport Faculty of Medicine, Technion - Israel Institute of Technology, Haifa, Israel. | Israel | HIC |
| PhD. | Laura | Piddock | Global Antibiotic R&D Partnership (GARDP), Geneva, Switzerland. | Switzerland | HIC |
| PhD. | Carolien | Ruesen | Centre for Epidemiology and Surveillance of Infectious Diseases, National Institute for Public Health and the Environment, Bilthoven, The Netherlands. | The Netherlands | HIC |
| PhD. | Maurizio | Sanguinetti | Dipartimento di Scienze Biotecnologiche di base, Cliniche Intensivologiche e Perioperatorie, Universitá Cattolica del Sacro Cuore, Rome, Italy. Dipartimento di Scienze di Laboratorio e Infettivologiche, Fondazione Policlinico Universitario A. Gemelli IRCCS, Rome, Italy. | Italy | HIC |
| MD. | Thorben | Schilling | University of Hohenheim, Institute of Animal Science, Department of Livestock Infectiology and Environmental Hygiene, Stuttgart, Germany. | Germany | HIC |
| DVM. | Remco | Schrijver | VetEffecT, Bilthoven, The Netherlands. | The Netherlands | HIC |
| MD. | Mitchell J. | Schwaber | Sackler Faculty of Medicine, Tel Aviv University, Tel Aviv, Israel. National Center for Infection Control, Israel Ministry of Health, Tel Aviv, Israel. | Israel | HIC |
| MD. | Luigia | Scudeller | FESCMID, Research and Innovation Unit, IRCCS Azienda Ospedaliero-Universitaria di Bologna, Bologna, Italy. | Italy | HIC |
| PhD. | Didem | Torumkuney | GlaxoSmithKline, Brentford, Middlesex, United Kingdom. | United Kingdom | HIC |
| PhD. | Thomas | Van Boeckel | ETH Zurich, Switzerland, Health Geography and Policy Group, Zurich, Switzerland | Switzerland | HIC |
| PhD. | Wannes | Vanderhaeghen | AMCRA, Center of expertise on Antimicrobial Consumption and Resistance in Animals, Brussels, Belgium. AACTING network. | Belgium | HIC |
| MD. | Andreas | Voss | Radboud University Medical Centre, Department of Medical Microbiology, Nijmegen, The Netherlands. | The Netherlands | HIC |
| PhD. | Teresa | Wozniak | Australian e-Health Research Centre CSIRO, Brisbane, Queensland, Australia. | Australia | HIC |

*Country income status - UMIC: Upper Middle Income Country, LMIC: Lower Middle Income Country, HIC: High Income Country (The World Bank. <https://datahelpdesk.worldbank.org/knowledgebase/articles/906519-world-bank-country-and-lending-groups>).

# Supplementary Table 2. List of the guidance documents publicly available and published from 2016 to 2021 providing indications on how antimicrobial resistance and antimicrobial consumption/residues data should be included in a One Health report.

| **Document title** | **Source and**  **year of publication** | **Sectors covered** | **Purpose** |
| --- | --- | --- | --- |
| [Global Tricycle Surveillance ESBL E. Coli](https://www.who.int/publications/i/item/who-integrated-global-surveillance-on-esbl-producing-e.-coli-using-a-one-health-approach) | WHO, OIE, FAO  2021 | Human  Animal  Environment | Define a multisector (human, animal and environment) surveillance system to detect and estimate the prevalence/quantity of the extended spectrum beta-lactamases- (ESBL) producing Escherichia coli. |
| [Technical brief on water, sanitation, hygiene, and wastewater management to prevent infections and reduce the spread of antimicrobial resistance](https://www.who.int/publications/i/item/9789240006416) | WHO, FAO, OIE  2020 | Human  Animal  Environment | Provide technical support to inform water, sanitation, and hygiene (WASH) and wastewater elements in national multi-sectoral antimicrobial resistance action plans. |
| [Taking a Multi-sectoral, One Health Approach: A Tripartite Guide to Addressing Zoonotic Diseases in Countries](https://www.fao.org/3/ca2942en/CA2942EN.pdf) | WHO, FAO, OIE  2019 | Human  Animal  Environment | Implement a One Health approach to treat zoonotic diseases and other common health threats at the human-animal-environment interface. |
| [Joint FAO/WHO Expert Meeting in collaboration with OIE on Foodborne Antimicrobial Resistance: Role of the Environment, Crops and Biocides](https://apps.who.int/iris/handle/10665/332387) | FAO, WHO  2019 | Human  Animal  Environment | Summarize the current scientific literature concerning the environmental transmission of antimicrobial-resistant bacteria and antimicrobial resistance genes. |
| [Monitoring and evaluation of the global action plan on antimicrobial resistance](https://apps.who.int/iris/handle/10665/325006) | WHO, FAO, OIE  2019 | Human  Animal  Environment | Provide a tool that can facilitate the production, collection and analysis of standardized data to inform policy decision on antimicrobial resistance strategies for the next 5–10 years at the national and global levels. |
| [Integrated Surveillance of Antimicrobial Resistance in Foodborne Bacteria](https://apps.who.int/iris/bitstream/handle/10665/255747/9789241512411-eng.pdf?sequence=1&isAllowed=y) | WHO  2017 | Human  Animal | Support the development of integrated antimicrobial resistance monitoring programmes in foodborne bacteria. |
| [Development and harmonisation of national antimicrobial resistance surveillance and monitoring programmes for aquatic animals (Chapter 6.4)](https://www.oie.int/fileadmin/Home/eng/Health_standards/aahc/2010/en_chapitre_antibio_development_harmonisation.htm) | OIE  2019 | Human  Animal | Establish criteria for the development of national antimicrobial resistance surveillance and monitoring programmes (or to harmonize existing one) in aquatic animals and aquatic animal products intended for human consumption. |
| [Harmonisation of national antimicrobial resistance surveillance and monitoring programmes (Chapter 6.8)](https://www.oie.int/fileadmin/Home/eng/Health_standards/tahc/current/chapitre_antibio_harmonisation.pdf) | OIE  2019 | Human  Animal | Establish criteria for the development of national antimicrobial resistance surveillance and monitoring programmes (or to harmonize existing one) in food-producing animals and in products of animal origin intended for human consumption. |
| [Role played by the environment in the emergence and spread of antimicrobial resistance (AMR) through the food chain](https://efsa.onlinelibrary.wiley.com/doi/epdf/10.2903/j.efsa.2021.6651) | EFSA  2021 | Animal  Environmental | Provide a scientific opinion on the role played by the environment in the emergence and spread of antimicrobial resistance through the food chain. |
| [Critically Important Antimicrobials for Human Medicine](https://apps.who.int/iris/bitstream/handle/10665/312266/9789241515528-eng.pdf) | WHO  2019 | Human  Animal | Promote the prudent use of antimicrobials in both human and veterinary medicine. |
| [Bridging the gap between surveillance data and antimicrobial stewardship in the animal sector – Practical guidance from the JPIAMR ARCH and COMBACTE MAGNET EPI-Net networks](https://www.jpiamr.eu/projects/arch/) | Journal of antimicrobial chemotherapy  2020 | Human  Animal  Environment | Define how to implement antimicrobial stewardship interventions taking in consideration local AMR surveillance data with a One Health approach in different settings. |
| [A conceptual framework for the environmental surveillance of antibiotics and antibiotic resistance](https://www.sciencedirect.com/science/article/pii/S0160412019304908?via%3Dihub) | Environment International Journal 2019 | Environment | Define key objectives for environmental AMR surveillance. |

# Supplementary Table 3. List of One Health surveillance reports published at least once between 2018 and 2021 by publicly-funded national or international surveillance systems.

| **One Health Surveillance report title** | **Link** | **Country** | **Reporting frequency** | **Language** | **Sectors included (Human/Animal/Envioronment** | **AMC /residues data coverage (Y/N)** | **AMR data coverage (Y/N)** | **Inclusion of comparative analysis/integrated analysis for AMC (Y/N/Not applicable)** | **Inclusion of comparative analysis/integrated analysis for AMR   (Y/N)** |
| --- | --- | --- | --- | --- | --- | --- | --- | --- | --- |
| Scottish One Health Antimicrobial Use and Antimicrobial resistance Annual report | https://www.hps.scot.nhs.uk/a-to-z-of-topics/antimicrobial-use-and-resistance/ | Scotland | Yearly | English | 3 (Human/Animal/Environment) | Y | Y | N | N |
| Nippon AMR One Health Report (NAOR) | <https://www.mhlw.go.jp/search.html?q=AMR+ONE+HEALTH+NIPPON+REPORT&cx=005876357619168369638%3Aydrbkuj3fss&cof=FORID%3A9&ie=UTF-8&sa=> | Japan | Yearly | English | 3 (Human/Animal/Environment) | Y | Y | N | N |
| Informe JIACRA españa: Primer análisis integrado del consumo de antibioticos y su relación con la aparición de resistencia | <https://www.resistenciaantibioticos.es/es/publicaciones/informe-jiacra-espana> | Spain | Yearly | Spanish | 2 (Human/Animal) | Y | Y | Y | N |
| Antimicrobial consumption and resistance in bacteria from humans and animals | <https://www.ema.europa.eu/en/veterinary-regulatory/overview/antimicrobial-resistance/analysis-antimicrobial-consumption-resistance-jiacra-reports> | European Union/European Economic Area | 3 years | English | 2 (Human/Animal) | Y | Y | Y | Y |
| The European Union Summary Report on Antimicrobial Resistance in Zoonotic and indicator bacteria from humans, animals and food | https://www.efsa.europa.eu/en/publications?s=The+European+Union+summary+report+on+antimicrobial+resistance+in+zoonotic+and+indicator+bacteria+from+humans | European Union/European Economic Area | Yearly | English | 3 (Human/Animal/Environment) | N | Y | n/a | N |
| Canadian Integrated Program for Antimicrobial Resistance Surveillance (CIPARS) | <https://www.canada.ca/en/public-health/services/surveillance/canadian-integrated-program-antimicrobial-resistance-surveillance-cipars.html> | Canada | Yearly | English | 2 (Human/Animal) | Y | Y | Y | Y |
| NARMS: National Antimicrobial Resistance Monitoring System | <https://www.fda.gov/animal-veterinary/national-antimicrobial-resistance-monitoring-system/narms-now-integrated-data> | United States of America | Yearly (interactive database) | English | 2 (Human/Animal) | N | Y | n/a | N |
| Sales of antibiotics and occurrence of antibiotic resistance in Sweden (SWEDRES/SVARM) | <https://www.sva.se/en/our-topics/antibiotics/svarm-resistance-monitoring/swedres-svarm-reports/> | Sweden | Yearly | English | 3 (Human/Animal/Environment) | Y | Y | Y | Y |
| Swiss Antibiotic Resistance Report (ANRESIS ARCH-Vet) | https://www.anresis.ch/publication-category/sarr/ | Switzerland | Annual (2 reports till date) | English | 3 (Human/Animal/Environment) | Y | Y | N | N |
| Usage of Antimicrobial Agents and Occurrence of Antimicrobial Resistance in Norway (NORM/NORM-VET) | <https://www.vetinst.no/en/surveillance-programmes/norm-norm-vet-report> | Norway | Yearly | English | 3 (Human/Animal/Environment) | Y | Y | N | N |
| UK One Health Report: antibiotic use and antibiotic resistance in animals and humans | https://www.gov.uk/search/research-and-statistics?parent=/health-and-social-care/antimicrobial-resistance&keywords=ONE%20HEALTH&content_store_document_type=all_research_and_statistics&topic=645d4bd6-84a2-49e0-bc38-f0d5459f583e&order=relevance | England & Wales | Annual (2 reports till date) | English | 2 (Human/Animal) | Y | Y | Y | Y |
| Resistenzbericht Österreich AURES: Antibiotikaresistenz und Verbrauch antimikrobieller Substanzen in Österreich | <https://www.sozialministerium.at/Themen/Gesundheit/Antimikrobielle-Resistenzen-und-Gesundheitssystem-assoziierte-Infektionen/Antimikrobielle-Resistenzen/AURES---der-%C3%B6sterreichische-Antibiotikaresistenz-Bericht.html> | Austria | Yearly | German | 3 (Human/Animal/Environment) | Y | Y | N | N |
| DANMAP: Use of antimicrobial agents and occurrence of antimicrobial resistance in bacteria from food animals, food and humans in Denmark | <https://www.danmap.org/reports> | Denmark | Yearly | English | 2 (Human/Animal) | Y | Y | N | Y |
| French National Observatory for Epidemiology of Bacterial Resistance to Antimicrobials (ONERBA), Annual report | <http://onerba.org/publications/rapports-onerba/> | France | Yearly | French | 2 (Human/Animal) | N | Y | n/a | N |
| Sýklalyfjanotkun og sýklalyfjanæmi baktería í mönnum og dýrum á Íslandi | https://www.landlaeknir.is/leitarnidurstodur/?q=S%C3%BDklalyfjanotkun+og+s%C3%BDklalyfjan%C3%A6mi+bakter%C3%ADa+%C3%AD+m%C3%B6nnum+og+d%C3%BDrum+%C3%A1+%C3%8Dslandi+ | Iceland | Yearly | Icelandic | 2 (Human/Animal) | Y | Y | N | N |
| Ireland: One Health Report on Antimicrobial Use & Antimicrobial Resistance | <https://www.gov.ie/en/publication/98f5bb-one-health-report-on-antimicrobial-use-antimicrobial-resistance/> | Ireland | First report | English | 2 (Human/Animal) | Y | Y | Y | N |
| NethMap: Consumption of antimicrobial agents and antimicrobial resistance among medically important bacteria in the Netherlands | https://swab.nl/en/nethmap-pvid369 | Netherlands | Yearly | English | 2 (Human/Animal) | Y | Y | N | Y |
| Informe de la resistencias antimicrobianas en bacteria zoonosicas e indicatores de personas, animales y alimentos | <https://www.mapa.gob.es/es/ganaderia/temas/sanidad-animal-higiene-ganadera/sanidad-animal/zoonosis-resistencias-antimicrobianas/default.aspx> | Spain | Yearly | Spanish | 2 (Human/Animal) | N | Y | n/a | N |

# Supplementary Table 4. Glossary of terms used across the text.

| **Term** | **Definition** | **Source** |
| --- | --- | --- |
| Antimicrobial resistance | The ability of a microorganism to be able to resist the action of an antimicrobial agent. This applies to all microorganisms in general, comprising fungi, bacteria, virus, and parasites. In the case of bacteria, the resistance impedes antibiotics to kill them or stop their growth. For the purpose of this report, antimicrobial resistance refers only to antibacterial agents. Antifungals and antivirals are not taken into consideration. | <https://www.ecdc.europa.eu/en/antimicrobial-resistance/facts/factsheets/experts> |
| Antimicrobial Use (AMU) | Data on antibiotics taken by the individual patients. Data are collected at the patient level, which allows a more comprehensive set of data to be gathered, such as information on indication, treatment schemes and patient  characteristics. The collection of data on antibiotic use requires more resources but provides additional information on prescribing practices, important for guiding antimicrobial stewardship activities. | <https://www.who.int/publications/i/item/who-report-on-surveillance-of-antibiotic-consumption> |
| Antimicrobial consumption  (AMC) | Estimates of aggregated data derived from import, sales, or reimbursement databases. These data, often collected for administrative purposes, are usually easily accessible and provide a proxy estimate of use of antimicrobials, for which data collection is often more laborious. | <https://www.who.int/publications/i/item/who-report-on-surveillance-of-antibiotic-consumption> |
| Antimicrobial residues | Concentrations of antimicrobials considered contaminations in the environmental matrices as they refer to a minimum level of concentration that may result in the development of antimicrobial resistance. | <https://doi.org/10.1016/j.envint.2020.105796> |
| Substandard and falsified antimicrobials | Substandard refers to authorised antimicrobials that fail to meet either quality standards or specifications, or both, while falsified refers to those that deliberately/fraudulently misrepresent their identity, composition, or source | <https://www.who.int/news-room/fact-sheets/detail/substandard-and-falsified-medical-products> |
| Denominator data, AMR | Lower proportion of a fraction. This is the total number of isolates tested or total number of the target population under surveillance/tested used to estimate proportion of resistance or frequency of AMR infection (e.g. per 100,000 tested patients), respectively. | <https://www.cdc.gov/csels/dsepd/ss1978/lesson3/section1.html> |
| Resistance interpretation guidelines | Established international or national guidelines such as Clinical and Laboratory Standards Institute (CLSI), European Committee for Antimicrobial Susceptibility Testing (EUCAST),  The British Society for Antimicrobial Chemotherapy (BSAC), are used for interpretation of antibiotic susceptibility test results. | <https://onlinelibrary.wiley.com/doi/pdf/10.1002/9781118675014.ch2> |
| Indicator | Quantitative and qualitative factor that provides a simple and reliable means to measure achievement, to reflect the changes connected to activities, or to help assess the performance of a programme or system. | <https://aginfra.d4science.org/web/orionknowledgehub/catalogue> |
| One Health | One Health is an integrated, unifying approach that aims to sustainably balance and optimize the health of people, animals and ecosystems.  It recognizes the health of humans, domestic and wild animals, plants, and the wider environment (including  ecosystems) are closely linked and inter-dependent.  The approach mobilizes multiple sectors, disciplines and communities at varying levels of society to work together to foster well-being and tackle threats to health and ecosystems, while addressing the collective need for clean water, energy and air, safe and nutritious food, taking action on climate change, and contributing to sustainable development. | <https://www.who.int/health-topics/one-health#tab=tab_1> |

# Supplementary Table 5. Definition of environment in current guidance documents.

| Definition | Source |
| --- | --- |
| The complex of physical, chemical, and biotic factors (e.g. climate, soil, living things) that act upon an organism or an ecological community and ultimately determine its form and survival; herein, this refers to the physical location and context in which people and animals live and interact. | Taking a Multi sectoral, One Health Approach: A Tripartite Guide to Addressing Zoonotic Diseases in Countries. World Health Organization (WHO), Food and Agriculture Organization of the United Nations (FAO) and World Organisation for Animal Health (OIE). 2019.  <https://www.who.int/publications/i/item/9789241514934> |
| Any location that is not within or on the human or domestic animal body and thus covered by human and animal surveillance efforts. | A conceptual framework for the environmental surveillance of antibiotics and antibiotic resistance. Huijbers, P., Flach, C. F., Larsson, D.. *Environment international*. 2019; 130: 104880.  <https://doi.org/10.1016/j.envint.2019.05.074>. |
| Environments where food of animal or non-animal origin is produced or processed, at both the pre-harvest (primary production) and post-harvest levels (processing: e.g. slaughterhouses, processing plants). Retail not included. | Role played by the environment in the emergence and spread of antimicrobial resistance (AMR) through the food chain. European Food Safety Authority. 2021. <https://www.efsa.europa.eu/en/efsajournal/pub/6651>. |
| Environment is all that which is external to the individual, including physical, biological, social, cultural and other factors. | ORION: One health suRveillance Initiative on harmOnization of data collection and interpretatioN. One Health EJP. 2018. <https://onehealthejp.eu/jip-orion/> |
